# An Eight-CircRNA Assessment Model for Predicting Biochemical Recurrence in Prostate Cancer

**DOI:** 10.3389/fcell.2020.599494

**Published:** 2020-12-10

**Authors:** Shuo Wang, Wei Su, Chuanfan Zhong, Taowei Yang, Wenbin Chen, Guo Chen, Zezhen Liu, Kaihui Wu, Weibo Zhong, Bingkun Li, Xiangming Mao, Jianming Lu

**Affiliations:** ^1^Department of Urology, Zhujiang Hospital of Southern Medical University, Guangzhou, China; ^2^Department of Urology, The First Affiliated Hospital of Jinan University, Guangzhou, China; ^3^Guangdong Key Laboratory of Urology, Department of Urology, Minimally Invasive Surgery Center, The First Affiliated Hospital of Guangzhou Medical University, Guangzhou Urology Research Institute, Guangzhou, China

**Keywords:** prostate cancer, circRNA, biochemical recurrence, immune infiltration, LASSO

## Abstract

Prostate cancer (PCa) is a high morbidity malignancy in males, and biochemical recurrence (BCR) may appear after the surgery. Our study is designed to build up a risk score model using circular RNA sequencing data for PCa. The dataset is from the GEO database, using a cohort of 144 patients in Canada. We removed the low abundance circRNAs (FPKM < 1) and obtained 546 circRNAs for the next step. BCR-related circRNAs were selected by Logistic regression using the “survival” and “survminer” R package. Least absolute shrinkage and selector operation (LASSO) regression with 10-fold cross-validation and penalty was used to construct a risk score model by “glmnet” R software package. In total, eight circRNAs (including circ_30029, circ_117300, circ_176436, circ_112897, circ_112897, circ_178252, circ_115617, circ_14736, and circ_17720) were involved in our risk score model. Further, we employed differentially expressed mRNAs between high and low risk score groups. The following Gene Ontology (GO) analysis were visualized by Omicshare Online tools. As per the GO analysis results, tumor immune microenvironment related pathways are significantly enriched. “CIBERSORT” and “ESTIMATE” R package were used to detect tumor-infiltrating immune cells and compare the level of microenvironment scores between high and low risk score groups. What’s more, we verified two of eight circRNA’s (circ_14736 and circ_17720) circular characteristics and tested their biological function with qPCR and CCK8 *in vitro*. circ_14736 and circ_17720 were detected in exosomes of PCa patients’ plasma. This is the first bioinformatics study to establish a prognosis model for prostate cancer using circRNA. These circRNAs were associated with CD8^+^ T cell activities and may serve as a circRNA-based liquid biopsy panel for disease prognosis.

## Introduction

Prostate cancer (PCa) is a high morbidity solid tumor most commonly detected in older males in developed countries (Siegel et al., 2020). Radical prostatectomy (RP) offers great therapeutic effect for most cases of localized PCa. Nonetheless, up to 50% patients will experience biochemical recurrence (BCR) after the operation (Freedland et al., 2005; Moris et al., 2020). Research has shown that the prognosis of prostate cancer depends on many factors, such as age, positive margin, pathological stage, Gleason score, PSA, and so on (Kreuz et al., 2020). Due to the heterogeneity of prostate cancer, the existing prognosis judgment system currently fails to meet the needs of every patient. There is an urgent need for a new prostate cancer prognosis prediction model to be constructed.

In the human genome, only 2% of genes are protein coding and up to 98% are non-coding RNAs. The discovery of non-coding RNAs has completely changed our understanding of cancer research (Slack and Chinnaiyan, 2019). The prognosis value of microRNA (miRNA) and long non-coding RNA (lncRNA) are widely reported in cancers (Yang et al., 2019; Mitra et al., 2020; Zhou et al., 2020). As the use of sequencing methods becomes more popular, new kinds of non-coding RNA are emerging. Circular RNA (circRNA) is a new category of non-coding RNA which has a covalent circular structure (Kristensen et al., 2019). By comparing them with other linear RNAs, circRNAs have been shown to exist widely in body fluids and are more difficult to degrade because of their framework (Su et al., 2019). What’s more, circRNAs are ideal biomarkers based on their tissue specific, stable, and easy to detect features (Salzman et al., 2013). The latest reports show that circRNAs play important roles in PCa progression, including proliferation, epithelial-mesenchymal transition (EMT), and apoptosis (Feng et al., 2019; Shen et al., 2020; Shi et al., 2020). Progressions in high-throughput sequencing methods and updated bioinformatics algorithms have facilitated research into circRNAs (Santer et al., 2019). However, there are no reports of prognostic models on circRNAs signature in PCa so far.

In our study, we screened high abundance BCR-related circRNAs from a cohort of 144 PCa patients and developed a new circRNA risk score model to predict the prognosis. The GO analysis of differentially expressed mRNAs between high and low risk score groups shows that the circRNAs may affect BCR using the tumor microenvironment (TME). What’s more, the following CIBERSORT and ESTIMATE analyses proves their effect in TME. In addition, we verified two circRNA’s (circ_14736 and circ_17720) characteristics and tested their biological function with qPCR and CCK8 *in vitro.* In summary, we are the first in constructing this circRNA risk score model to predict the prognosis of PCa, which may be helpful in guiding therapeutic strategy.

## Materials and Methods

### Ethics Statement

This study was approved by the Ethics Committee of Zhujiang Hospital, Southern medical University. Informed consent forms were signed by all patients. According to the ethical and legal standards, three blood samples from PCa patients were handled and made anonymous.

### Dataset Preparation

circRNA sequencing data and corresponding clinical follow-up information were downloaded from the Gene Expression Omnibus (GEO) database^1^. We obtained the GSE113124 (Chen et al., 2019) expression profiles, including 144 patients with prostate cancer, for the next analysis by removing the circRNAs of FPKM (Fragments Per Kilobase Million) < 1 from expression dataset. The clinical characteristics of the patients are summarized in Table 1.

**TABLE 1:** Clinical characteristics of the prostate cancer patients.

**Parameter**		**GSE113124 (*n* = 144)**	
**Age at diagnosis (mean ± *SD*)**		**61.5 ± 6.5**	
Clinical stage, *n* (%)	≤T1c	83	57.6%
	≥T2a	61	42.4%
Gleason score, *n* (%)	≤6	11	7.6%
	=7	123	85.4%
	≥8	9	4.2%
	Null	1	0.7%
PSA at diagnosis (ng/ml), *n* (%)	0–3.9	17	11.8%
	4–9.9	93	64.6%
	≥10	34	23.6%
BCR, *n* (%)	Yes	30	20.8%
	No	114	79.2%
Metastasis, *n* (%)	Yes	9	6.3%
	No	134	93.1%
	Null	1	0.7%

### Construction of LASSO Model

circRNAs with FPKM > 1 were analyzed the Logistic regression; *P* < 0.05 was used as the significance cut-off to identify candidate circRNAs associated with BCR and the median of sample expression was used as the cutoff of high and low expression groups. Twenty eight circRNAs were selected, and the least absolute shrinkage and selector operation (LASSO) regression (Ramsay et al., 2018) with 10-fold cross-validation and penalty was used to construct a prognostic prediction model by “glmnet” R software package. Patients of the GSE113124 dataset were randomly assigned to 10 groups; one of these was the test set and the remaining nine groups were the training set. The risk score was calculated by expression profile data and the coefficient of the corresponding circRNAs. The formula is as follows:

$$R\,i\,s\,k\,S\,c\,o\,r\,e\,\left(R\,S\right)=\sum_{i=1}^{n}C\,o\,e\,f\,\left(i\right)\,X\,\left(i\right)$$

where *n* is the number of circRNAs in the prognostic prediction model, Coef(i) represents the coefficient, and X(i) means the relative circRNAs expression level identified by LASSO regression.

### Assessment of the Prognostic Prediction Model

Kaplan-Meier method was employed to draw the survival curves and assess the BCR of high and low risk score groups. Receiver operating characteristic (ROC) curve from “pROC” R package was performed to assess the sensitivity and specificity of the model compared with PSA, Gleason score, and pathologic stage. We identified differentially expressed mRNAs between different risk-score groups with FDR < 0.05 and —log2FoldChange— > 1, and Gene Ontology (GO) pathway analysis of those mRNAs were performed using OmicShare online tool. The background gene list is all Homo sapiens genes with Ensembl release 96 and Ensembl Genomes 43.

### Nomogram Construction

The Cox proportional hazard model for 5-year BCR free probability were fit to 144 patients by an “rms” R package, and the variables included PSA, clinical T stage, Gleason score, risk-score, and C-index of the nomogram.

### Immune Analysis by CIBERSORT and ESTIMATE

To calculate the tumor-infiltrating immune cells (TIICs) in the tumor samples, we used normalized gene expression profiles and LM22 signature matrix at 1,000 permutations to run the CIBERSORT^1^, which is a deconvolution algorithm website. Then the result of CIBERSORT was employed to draw a boxplot of immune cells based on risk-score group. For comparing the level of microenvironment scores between high and low risk groups, we obtained all RNA expression profiles by annotating with GRCh37. The result of running the ESTIMATE package (Yoshihara et al., 2013) was conducted to study the difference of microenvironments between the two groups.

### Cell Lines and Cell Culture

The prostate cancer cell lines PC3, DU145, C4-2, and LNCaP, were all purchased from Beina Biotechnology Research Institute. PC3 and DU145 were cultured in DMEM medium. C4-2 and LNCaP were cultured in RPMI-1640 medium. Both DMEM and RPMI-1640 medium were supplemented with 10% fetal bovine and 1% Penicillin and Streptomycin. The immortalized prostate epithelial cell line RWPE-1 was recovered from the nitrogen liquid tank in our lab and was maintained in KM medium with 1% recombinant epithelial growth factor. All the cell lines above were cultivated in the incubator with constant 5% CO_2_ at 37°C.

### RNase R Treatment, RT-qPCR, and Agarose Gel Electrophoresis

1 × 10^6^ cells were prepared for total RNA extraction with Trizol reagent. RNA from cytoplasmic and nuclear sections were separated according to the manufacturer of PARIS^TM^ Kit (Life technologies). To acquire pure circRNA, total RNA was digested by RNase R (Rev: 20180502; Geneseed) at 37°C for 10 min. Then, RNA was transcribed into cDNA with HiScript II Q RT SuperMix for qPCR (R223-01, Vazyme). SYBR Green real-time PCR Master Mix (QPK-201, TOYOBO) was used to assess the expression level of target circRNAs by Bio-Rad CFX96 PCR machine. β-actin was used as the inner reference. Finally, the PCR product was separated by 1.5% agarose gel electrophoresis to detect the existence and molecule weight of each targeted gene. Convergent and divergent primers of circ_14734, circ_17720, and GAPDH are shown in Supplementary Table 1.

### SiRNA Transfection and Cell Proliferation Assay

All small interference RNAs (siRNA) were synthesized by Hippo Biotechnology Company. DU145 and PC3 cells were seeded into the 6-well plates, then the negative control siRNA and siRNAs targeted to objective genes mixed with lipofectamine 3000 (L3000008, ThermoFisher) were added to the prepared cells in the fusion of 60–70%. RT-qPCR was used to detect the knockdown efficiency in DU145 and PC3 cells. Cell proliferation assay was carried out with CCK-8 kit (C0042, Beyotime Biotechnology),and cell viability was evaluated by the absorbance at 450nm using the microplate reader (Bio-Rad iMark). More details can be found in our previous study (Mo et al., 2017). SiRNAs sequence targeted to circ_14734 and circ_17720 are shown in Supplementary Table 1.

### Exosome Extraction and Identification

Before exosome isolation, the cultured cells were maintained in exosome free medium for 24 h. In brief, 10 ml culture medium with exosome free FBS was collected and centrifuged at 1,000 rpm for 10 min. Then the supernatant was filtrated with 0.22 μm filter to remove the cell debris. Subsequently, the medium containing exosomes was incubated with one quarter volume of PEG (polyethylene glycol) 8000 buffer overnight at 4°C (Sun et al., 2019). Finally, the mixture was centrifuged at 3,000 rpm for 30 min and re-suspended with 20 μl PBS. The morphology of exosomes was detected by Transmission Electron Microscope (TME), and the particle size distribution was examined via NanoSight. The exosome markers, including HSP70 (Proteintech, No.: 10995-1-AP) and CD63 (Proteintech, No.: 25682-1-AP), were examined by Western blot, and the details of this procedure can be found in our previous study (Mo et al., 2017).

### Statistical Analysis

R (version 3.6.1)^3^ and related packages were applied to all statistical analyses. The Wilcoxon test was used to compare two independent non-parametric samples. The Kaplan-Meier survival curves were verified with the log-rank test. We used mean ± standard deviation to describe the continuous variables in normal distribution while the median (range) was applied to testify the continuous variables in abnormal distribution. *P* < 0.05 was regarded as statistically significant.

## Results

### Constructing a CircRNA’s Signature Model to Predict BCR in 144 Patients With Prostate Cancer

**FIGURE 1:**
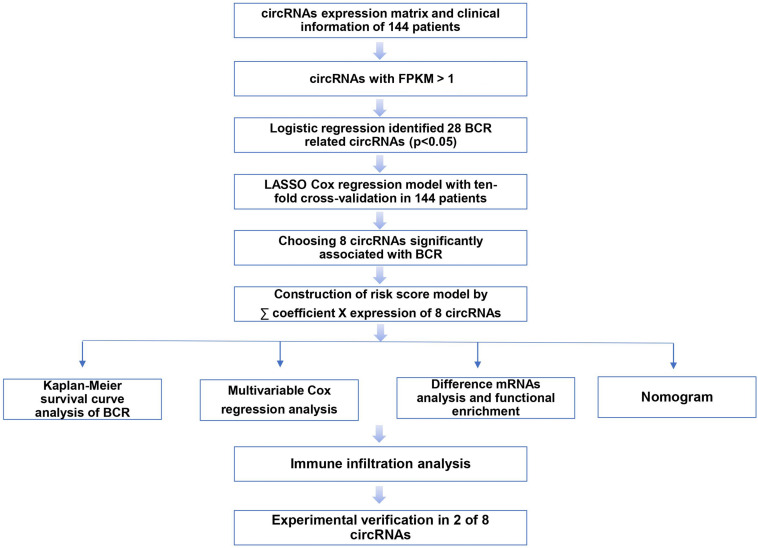
The flow diagram of this study.

**FIGURE 2:**
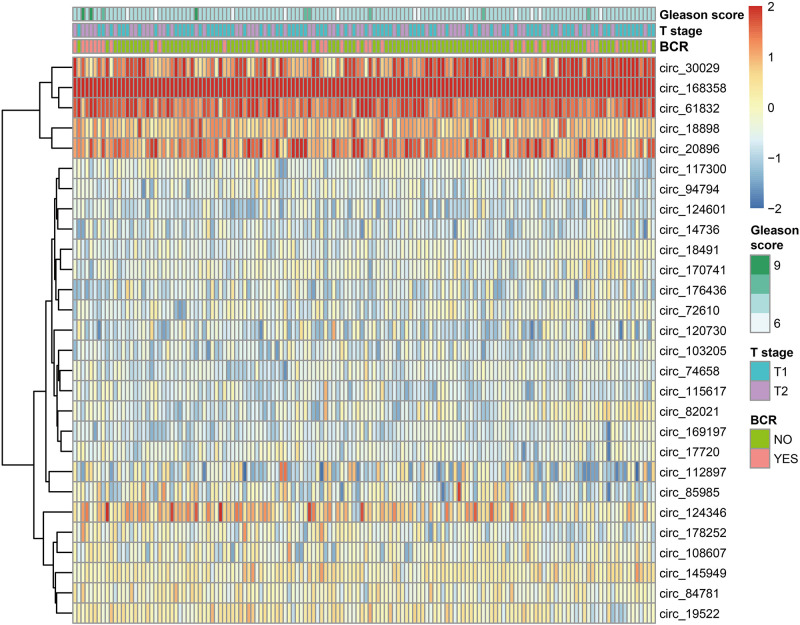
The heatmap of 28 BCR-related circRNAs based on logistic regression.

**FIGURE 3:**
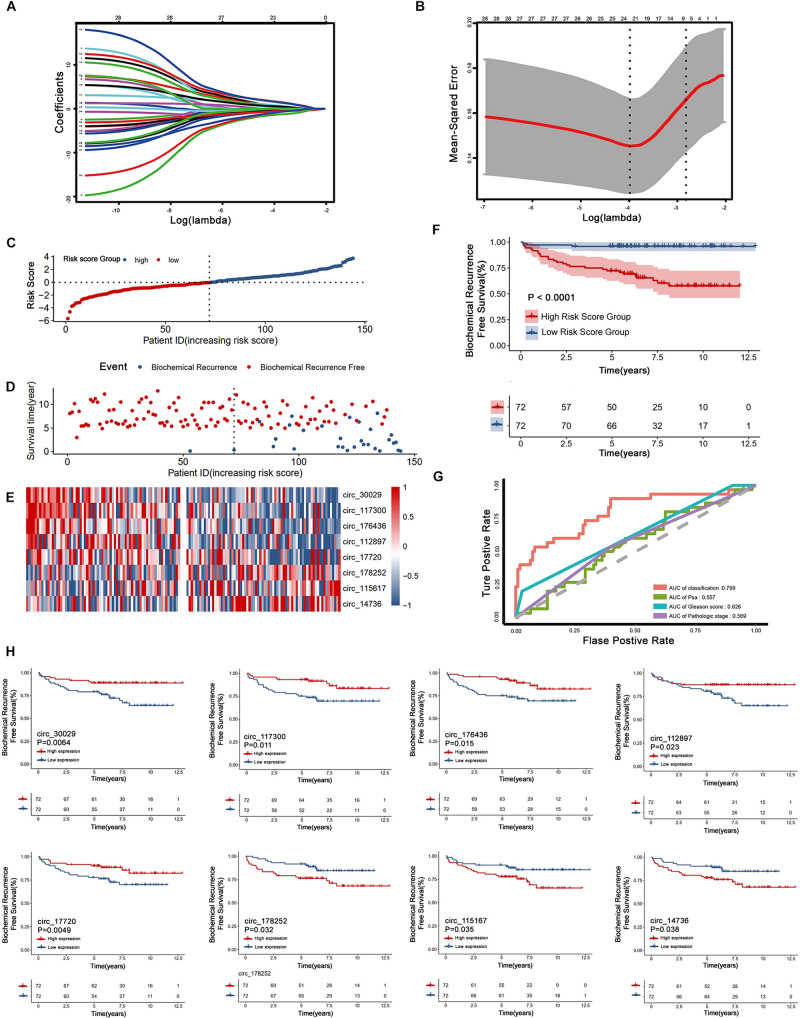
Construction and assessment of the eight circRNAs signature associated with biochemical recurrence by LASSO regression. **(A)** Process of variable selection in LASSO regression with 10-fold cross-validation. **(B)** Confidence interval in every lambda of LASSO regression. **(C–E)** circRNA predictor-score analysis of 144 PCa patients. The horizontal axis represents the 144 patients. Heatmap of circRNA expression level. **(F)** Biochemical recurrence analysis for the classifier. **(G)** Time-dependent ROC analysis curve for the classifier and clinical factors. **(H)** Kaplan-Meier estimates of eight circRNAs signature associated with biochemical recurrence.

Our workflow is showing in Figure 1. By analyzing expression profiles of circRNA in 144 PCa patients, the filter with mean FPKM for circRNAs less than 1 were identified 546 circRNAs from GSE113124. To study the relationship between circRNAs and biochemical recurrence, those circRNAs were subjected to logistic regression with low or high expression levels of each circRNA as the variable. Using a *P*-value of 0.05 as the cut-off criterion for circRNAs selection, we obtained 28 circRNAs that were significantly linked to biochemical recurrence. The expressions of those 28 circRNAs are shown as a heatmap in Figure 2. Target circRNAs were selected and based on a LASSO regression model. The best model was determined by L1 regularization and 10-fold cross-validation with the AUC (Area Under Curve) as the benchmark (Figures 3A,B). A range of models was constructed for selecting circRNAs. The best model (AUC value = 0.923) incorporating eight circRNAs (circ_30029, circ_117300, circ_176436, circ_112897, circ_178252, circ_115617, circ_14736, and circ_17720) was identified.

Non-zero coefficients and the expression of eight circRNAs in the LASSO regression model constructed the following risk-score formula for BCR prediction: Risk score = (-0.0637^∗^expression level of circ_30029) + (-0.0991 ^∗^expression level of circ_117300) + (-0.0015^∗^expression level of circ_176436) + (-0.0185^∗^expression level of circ_112897) + (-0.0187^∗^expression level of circ_17720) + (0.0809 ^∗^expression level of circ_115617) + (0.0027 ^∗^expression level of circ_14736) + (0.0906 ^∗^expression level of circ_178252). The essential information and Kaplan-Meier plots of eight circRNAs were shown in Table 2 and Figure 3H. Three circRNAs with positive coefficients (circ_178252, circ_115617, and circ_14736) laid out high expression with an abhorrent outcome. Meanwhile, the negative coefficients in the remnants (circ_30029, circ_117300, circ_176436, circ_112897, and circ_17720) indicated high expression was correlated with less biochemical recurrence.

**TABLE 2:** CircRNAs significantly associated with the biochemical recurrence free survival in the test series patients (*N* = 144).

**CircRNA**	**Position**	**Length**	**Coefficient**	**Gene symbol**	**Description**	**Associated diseases of parent gene**	**Associated diseases of circRNA**
circ_30029	chr2:168920009-168986268: -	402	−0.0637	STK39	Serine/threonine kinase 39	Lung cancer (Li et al., 2016) Hypertension (Jamshidi et al., 2018)	–
circ_117300	chr7:131071878-131084192: +	535	−0.0991	MKLN1	Muskelin 1	Childhood asthma (Ding et al., 2013)	Active tuberculosis (Huang et al., 2018b)
circ_176436	chr6:160819010-160831878: +	546	−0.0015	SLC22A3	Solute carrier family 22 member 3	Colorectal cancer (Ren et al., 2019)	Ovarian cancer (Zhang et al., 2020b)
circ_112897	chr16:20636744-20638638: -	161	−0.0185	ACSM1	Acyl-CoA synthetase medium	Breast carcinoma (Celis et al., 2008)	–
					Chain family member 1	Depressive disorder (Li W. et al., 2015)	
circ_178252	chr8:62593526-62596747: -	264	0.0906	ASPH	Aspartate beta-hydroxylase	Liver cancer (de la Monte et al., 2006)	–
						Lung carcinoma (Luu et al., 2009)	
circ_115617	chr4:87693930-87696805: +	723	0.0809	PTPN13	Protein tyrosine phosphatase non- receptor type 13	Colorectal cancer (Laczmanska et al., 2017)	–
						Breast cancer (Glondu-Lassis et al., 2010)	
circ_14736	chr1:58971731-59002413: -	865	0.0027	OMA1	OMA1 zinc metallopeptidase	Gynecologic cancers (Kong et al., 2014)	–
circ_17720	chr1:8555122-8674745: -	708	−0.0187	RERE	Arginine-glutamic acid dipeptide repeats	Leukemia (Waerner et al., 2001)	–

**FIGURE 4:**
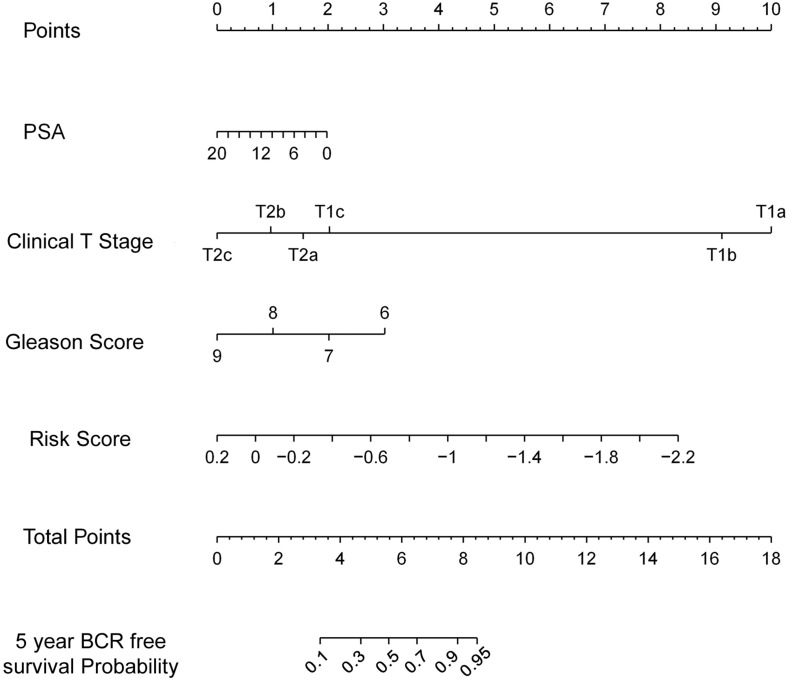
The nomogram to predict likelihood of 5-year BCR-free survival. Nomogram predicting PCa patients’ 5-year BCR-free survival. Each variable is projected to the point scale to obtain a value. The higher the value, the more likely to have BCR.

Figures 3C–E shows the relation among prognostic scores, the biochemical recurrence status, and prostate cancer circRNAs expression in 144 patients ranked by the prognostic score of the eight-circRNAs signature. Patients were divided into high-risk score group and low-risk score group by the median prognostic risk score. The higher risk scores revealed more BCR and less BCR-free time in PCa patients. Kaplan-Meier survival curve shows that patients with high-risk scores have statistically higher BCR than that of the low-risk score group (Figure 3F). As shown in Figure 3G, time-dependent ROC curve analyses were conducted to illustrate the sensitivity and specificity of BCR prediction. The AUC for the eight circRNAs signature prognostic model (AUC = 0.799) was calculated and compared with the AUC of clinical factors (AUC of PSA = 0.557, AUC of Gleason score = 0.626 and AUC of pathological stage = 0.569). Then, multivariate Cox regression analysis was carried out to estimate the prognostic value of the signature on BCR-free survival. With Gleason score, PSA, and pathological stage as covariates, the result shows that the eight-circRNAs signature was an independent prognostic factor for prostate cancer patients (Table 3). What’s more, we constructed a nomogram to predict the 5-year BCR-free survival rate of prostate cancer patients by the risk score and clinical factors, and the C-index of nomogram was 0.796 (Figure 4).

**TABLE 3:** Cox proportional hazards models in 144 patients.

**Factor**	**Multivariable**	
	**HR (95% CI)**	***P***
Eight-circRNAs classifier	11.68(3.45 to 39.57)	<0.001^∗^
Gleason score	2.41 (0.92 to 6.31)	0.072
PSA	1.48 (0.45 to 4.92)	0.522
Pathological stage	2.23 (1.03 to 4.80)	0.042^∗^

*Two-sided likelihood ratio test. CI, confidence interval; HR, hazard ratio.

### The Link Between 8 CircRNA’s Signature and Tumor Microenvironment

**FIGURE 5:**
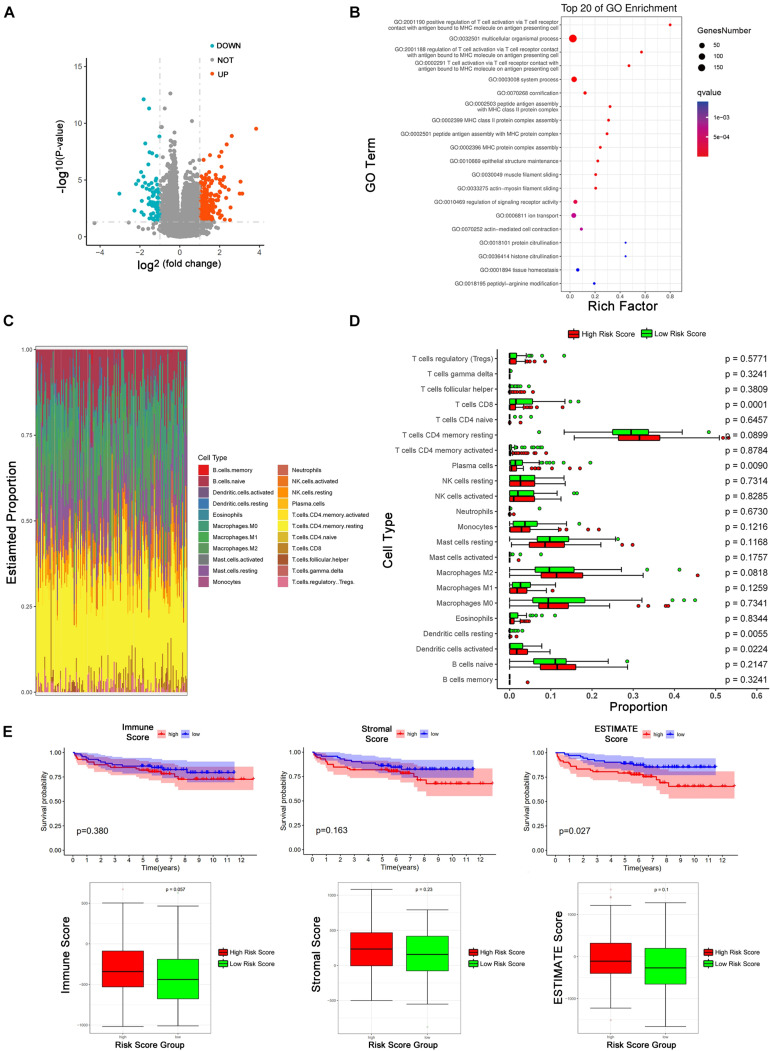
Tumor immune microenvironment between low-risk and high-risk score group. **(A)** Volcano Plot shows the differentially expressed mRNAs between high and low risk score groups (The cutoff value is —logFC— > 1 and *p* < 0.05). **(B)** GO pathways analysis of differentially expressed mRNAs. **(C)** The proportion of 22 kinds of immune cells in individual. **(D)** The proportion of 22 immune cells between high and low risk score groups. The red means high risk score and the green represents low risk score. The parameter *p*-value was obtained by Wilcoxon test. **(E)** Kaplan-Meier analysis of BCR and the comparison of different risk-score group based on immune score, stromal score, and estimate score from ESTIMATE package.

We employed DESeq2 to work out the differentially expressed mRNAs between the high risk score and low risk score groups. 338 differentially expressed mRNAs were enriched (logFC > 1 or logFC < -1, adjust *P* < 0.05), including 261 up-regulated mRNAs and 77 down-regulated mRNAs (Figure 5A). We presented the top 20 enriched GO terms (*P* < 0.05) by GO analyses involving biological process (BP), molecular function (MF), and cellular component (CC) in Figure 5B, which also includes the positive regulation of T cell activation via T cell receptor contact with antigens bound to MHC molecules on antigen presenting cells, MHC class II protein complex assembly, and MHC protein complex assembly.

Emerging evidence revealed that circRNAs related to the tumor microenvironment, involved in roles such as tumor surveillance, endothelial monolayer permeability, angiogenesis, hypoxia, remodeling of the extracellular matrix (ECM), exosomes, and so on (Ou et al., 2019; Hu et al., 2020; Huang et al., 2020). Based on the results of the GO analysis, we explored our eight circRNAs signature in the tumor immune microenvironment. CIBERSORT was employed to estimate the abundances of 22 immune cell types using mRNA expression data in GSE 113124 (Figure 5C). In Figure 5D, patients in the low-risk group had more CD8^+^ T cells (*P* < 0.001), plasma cells (*P* <0.01), and dendritic cells resting (*P* <0.01), and patients in the high-risk group had more activated dendritic cells (*P* <0.05). ESTIMATE R package was used to obtain immune score, stromal score, and ESTIMATE score of patients. According to medians of the three scores, patients were divided into a high score group and low score group. KM curves describe the relationships between biochemical recurrence and immune score, stromal score, and ESTIMATE score (Figure 5E). In ESTIMATE evaluation, it has a consistent trend with CIBERSORT that shows a high-risk score is positively correlated with high immune infiltration, although there is no significant statistical difference.

### Experimental Verification of Circ_14734 and Circ_17720

**FIGURE 6:**
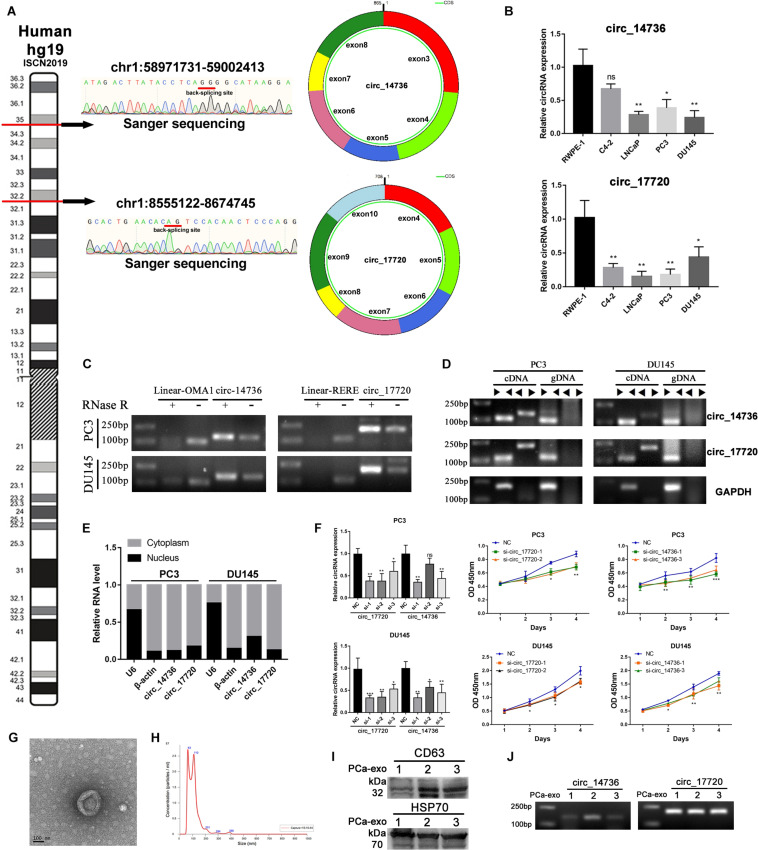
Experimental validation of circ_14736 and circ_17720. **(A)** The back-splicing junction site of circ_14734 and circ_17720 was determined by sanger sequencing. **(B)** The naive expression of circ_14734 and circ_17720 in prostate cancer cell lines. **(C)** circ_14734 and circ_17720 could be detected after the treatment of RNase R digestion. **(D)** the amplification of circ_14734 and circ_17720 by convergent and divergent primers was examined in cDNA and gDNA. **(E)** The distribution of circ_14734 and circ_17720 by nuclear and cytoplasmic fraction was determined by RT-qPCR. **(F)** Si-RNAs knock down effect screening. circ_14734 and circ_17720 inhibited DU145 and PC3 cell proliferation via CCK-8 assay. **(G,H)** The morphology and particle size distribution of Plasma-derived exosomes were verified by TEM and Nano-Sight. **(I)** Western blot analysis of exosomes isolated from three PCa patients’ blood plasma. The exosomal markers CD63 and HSP70 were detected. J. circ_14734 and circ_17720 were examined in blood plasma exosomes by gel electrophoresis.

According to a pervious study, circ_14734 and circ_17720 can be detected in PCa patients’ urine (Vo et al., 2019). We wondered if they had potential to be biomarkers. What’s more, we further characterized these two circRNAs by experimental analysis. Firstly, we specially designed the divergent primers targeted to each selected circRNA and identified the circularized site through Sanger sequencing (Figure 6A). Then we examined the basal expression of circ_14734 and circ_17720 through RT-qPCR, and found both to be downregulated in PCa cell lines (Figure 6B). Circ_14734 and circ_17720 were all resistant to RNase R digestion, while the linear mRNA of their parent genes were not (Figure 6C). Additionally, we found that circ_14734 and circ_17720 could be amplified only in cDNA instead of in gDNA (Figure 6D), which indicates that circ_14734 and circ_17720 were not the results of trans-splicing or genomic rearrangements (Jeck and Sharpless, 2014). Moreover, to further obtain the cell distribution of circ_14734 and circ_17720, cytoplasmic and nuclear fraction assay and RT-qPCR were conducted, and the results showed that circ_14734 and circ_17720 were mainly located in the cytoplasm of DU145 and PC3 cells (Figure 6E). Finally, to explore the function of circ_14734 and circ_17720, oligo RNAs containing si-NC and siRNAs specially targeting to the back-splicing junction site of circ_14734 and circ_17720 were transfected into DU145 and PC3 cells. It was confirmed that both first two siRNAs that targeted circ_14734 and circ_17720 efficiently knocked down their expression at around 70% via RT-qPCR. Then, CCK-8 assay was applied to detect the cell viability of NC and knockdown groups. As shown in Figure 6F, knockdown of circ_14734 or circ_17720 both inhibited the proliferation of DU145 and PC3 cells.

Considering circ_14734 and circ_17720 were closely related to immune infiltration, which was regarded as a sort of tumor microenvironment, we began to consider whether there was a potential connection between tumor-cell-derived exosomes and the immune microenvironment. Therefore, we collected exosomes from serum in the manner of precipitation with PEG 8000 and verified the morphology and particle size through TME and NanoSight. The results showed that the exosomes were nearly spherical, and the particle size mainly distributed in around 100 nm (Figures 6G,H). CD63 and HSP70, two exosomal markers, were detected by western blot in three PCa patients’ blood plasma (Figure 6I). Subsequently, RT-qPCR and Agarose gel electrophoresis were performed to testify the existence of circ_14734 and circ_17720, and the results revealed that both circ_14734 and circ_17720 could be detected in exosomes derived from three PCa patients (Figure 6J).

## Discussion

PCa remains one of the most common malignant tumors in men (Welch and Albertsen, 2020). Generally, BCR is a useful clinical index to monitor disease progression (Fenton et al., 2018). As reported by the European guidelines, BCR is characterized as two consecutive PSA ≥ 0.2 ng/ml after surgical procedure (Mottet et al., 2020). Moreover, some BCR cases are going to evolve into metastasis, which means a worse prognosis (Dall’Era et al., 2012). Due to the complex molecular mechanism of development and progression, the existing methods of PCa prognosis prediction are still deficient. We need to develop some prediction models for early detection, individualized therapeutic strategy, and distinguishing high-risk patients.

Molecular signatures have been related to predict the prognosis in some kinds of tumors. The risk score model based on non-coding RNAs, such as miRNAs and lncRNAs, has been largely reported in PCa (Pang et al., 2019). Through miRNA analysis in 78 PCa blood samples, Hoey et al. (2019) identified four circulating miRNAs which could accurately stratify patients into high- and low-risk categories after radical prostatectomy. In addition, Shao et al. (2019) built up a prognostic model using seven lncRNA signatures and revealed that the high risk of this model predicted faster BCR of PCa. However, the prognosis value of circRNAs in PCa is still not clear. Compared with other non-coding RNAs, circRNAs have a typical circular structure that resist the degradation of RNase and could be easier detected in body fluid (Su et al., 2019). There is growing research about the dysregulation of circRNAs in cancer. For example, Chen et al. (2020) found that circ-MALAT could directly bind to ribosome and PXA5 mRNA, forming a ternary complex, and functioned as a miRNAs sponge to sustain the self-renewal of hepatocellular cancer stem cells. Additionally, it has been reported that circASPA1 promoted hepatocellular carcinoma (HCC) progression by regulating miR-326/miR-532-5p-MAPK1 signaling, and positively correlated with tumor-associated-macrophage infiltration via the miR-326/miR-532-5p-CSF-1 axis (Hu et al., 2019). Moreover, circRNAs also exert an influence on metabolic reprogramming in malignant tumors. ciRS-122, which was confirmed to be an exosome-transmitted circRNA, could promote glycolysis to strengthen chemoresistance colorectal cancer cells (Wang et al., 2020).

Our study is the first to report a circRNAs signature model in predicting the BCR of PCa. In our work, the mRNAs and circRNAs obtained based on high-throughput sequencing and clinical data of 144 PCa patients are from GSE113124. Firstly, 28 circRNAs whose FPKM was more than 1.0 and were significantly linked to biochemical recurrence in logistic regression were selected. Subsequently, LASSO regression and 10-fold cross-validation targeted eight circRNAs to construct a best risk score model (AUC value = 0.923) for predicting BCR. Our eight circRNAs risk score model (circ_30029, circ_117300, circ_176436, circ_112897, circ_178252, circ_115617, circ_14736, and circ_17720) shows that patients with high-risk scores are statistically significantly higher than the low-risk score group in the rate of BCR by KM survival curve. What’s more, in the ROC and multivariate Cox regression analysis, the prognostic value of our risk score model is better than the existing clinical indexes.

Among the eight circRNAs in our model, only a few have been reported in medical research. Circ_117300 may be specifically found in tuberculosis patients’ plasma (Huang et al., 2018). In addition, circ_176436 inhibited ovarian cancer progression by suppressing miR-518a-5p to induce Fas expression (Zhang et al., 2020). To assess their function in PCa, we gathered the differentially expressed mRNAs between high and low risk score groups. Some T cell and MHC related pathways have been enriched in the GO analysis. As we know, T cells and MHC are two important parts of the immune system, so we hypothesized that these eight circRNAs could affect the tumor microenvironment in prostate cancer. As per the results of our following CIBERSORT and ESTIMATE analyses, we proved our assumption. In the CIBERSORT analysis, CD8^+^ T cells were markedly decreased in the high-risk score group. CD8^+^ T cells act as cytotoxic T cells to anti-tumor in most conditions (Qiao et al., 2019; Lynn et al., 2020). For our study, the eight circRNAs may promote BCR by protecting PCa cells as a result of cut down of the CD8^+^ T cells in the high-risk score group. Coincidentally, we have a consistent trend that the high-risk score is positively correlated with the high immune infiltration in ESTIMATE analysis, although there are no significant statistical differences.

To verify the individual capacity of these circRNAs, we selected circ_14736 and circ_17720 in our experimental validation because they exist in urine. We identified their junction sites by Sanger sequencing, examined the native expression in prostate cell lines, and characterized their circularized structure. What’s more, circ_14736 and circ_17720 showed cell viability inhibition effects in CCK8 assay. Finally, we detected them in the exosomes of PCa patients’ plasma. It gave us some hints that we could develop their prognosis values by blood test. To date, only a minor part of circRNAs’ biological functions have been identified, with most of them playing a miRNA sponge role (Hansen et al., 2013; Memczak et al., 2013). In addition, immunoprecipitation experiments data showed that circRNAs can interact with proteins directly (Ashwal-Fluss et al., 2014; Li Z. et al., 2015; Du et al., 2016; Abdelmohsen et al., 2017; Chen et al., 2018), through roles such as decoy proteins, enhancing protein function, protein scaffolding, and recruiting protein. Interestingly, some circRNAs have a protein or polypeptide translation ability (Legnini et al., 2017; Yang et al., 2018; Zhang et al., 2018), although most circRNAs are regarded as non-coding. In our data, circ_14736 and circ_17720 are mainly located in cytoplasm. What’s more, they both have a binding site with miRNAs and proteins according to the circinteractom database. Furthermore, only circ_17720 revels a protein coding potential (Fickett score: 1.1294, Hexamer score: 0.1549, IRES elements score: 0.90). We will explore the function and mechanism of circ_14736 and circ_17720 in our next study.

Unfortunately, due to the limitation of our clinical samples’ amount and patients’ prognosis information, we cannot verify our model in different datasets. We will monitor any updated circRNAs datasets to improve the accuracy of our model. We are also going to collect more samples to develop their prognosis value in blood.

## Conclusion

We constructed an eight circRNAs risk score model to reliably predict the BCR of PCa patients. We found that the BCR predicting effect may be related to the tumor microenvironment. At the same time, we preliminarily verified the function of circ_14736 and circ_17720 *in vitro*. Further experiments are necessary to clarify their roles in PCa.

## Data Availability Statement

The original contributions presented in the study are included in the article/supplementary material, further inquiries can be directed to the corresponding author/s.

## Ethics Statement

The studies involving human participants were reviewed and approved by the Ethics Committee of Zhujiang Hospital, Southern medical University. The patients/participants provided their written informed consent to participate in this study.

## Author Contributions

JL and XM designed the study. SW and WS analyzed the data. CZ did the experimental validation. TY, WC, GC, ZL, KW, and WZ searched literature. JL and BL wrote the manuscript. All authors read and approved the manuscript.

## Conflict of Interest

The authors declare that the research was conducted in the absence of any commercial or financial relationships that could be construed as a potential conflict of interest.

## Footnotes

1 http://www.ncbi.nlm.nih.gov/geo/

1 http://cibersort.stanford.edu/

3 https://www.r-project.org/

## Abbreviations

AUC: Area Under Curve
BCR: Biochemical recurrence
BP: Biological Process
CC: Cellular Component
cDNA: Complementary DNA
circRNAs: Circular RNAs
DMEM: Dulbecco’s Modified Eagle’s medium
DU145: Duke University 145
ECM: Extracellular matrix
EMT: Epithelial-mesenchymal transition
FBS: Fetal bovine serum
FPKM: Fragments Per Kilobase Million
gDNA: Genomic DNA
GEO: Gene Expression Omnibus
GO: Gene Oncology
HCC: Hepatocellular carcinoma
HGNA: HUGO Gene Nomenclature Committee
KM survival curve: Kaplan-Meier survival curve
LASSO: Least absolute shrinkage and selection operator
LNCaP: Lymph Node Carcinoma of the prostate
lncRNA: Long non-coding RNA
MF: Molecular Function
MHC: Major histocompatibility complex
miRNA: MicroRNA
PCa: Prostate cancer
PSA: Prostate-specific antigen
Rnase R: Ribonuclease R
ROC: Receiver-operating characteristic
RP: Radical prostatectomy
RT-qPCR: Quantitative reverse transcription PCR
siRNA: Small interference RNAs
TIICs: Tumor-infiltrating immune cell
TME: Tumor microenvironment.
