# Supplementary material for: An Eight-CircRNA Assessment Model for Predicting Biochemical Recurrence in Prostate Cancer
Source: Front Cell Dev Biol. 2020 Dec 10;8:599494. doi: 10.3389/fcell.2020.599494 (PMC7758402; doi:10.3389/fcell.2020.599494)
Supplement: Supplementary file 1 [file Table_1.docx]

| Primers | | |
| --- | --- | --- |
| Divergent primers |  | 5'-3' |
| OMA1 | Forward | GGCAGCAAATGGAGTTCGTT |
|  | Reverse | TTCTTGTTAGGAGGAAGTGCC |
| RERE | Forward | TGGATGCCTGGAGTTAACGA |
|  | Reverse | GGTCACACAAAGCAGGAGTT |
| GAPDH | Forward | TGCCATGTAGACCCCTTGAA |
|  | Reverse | GTCCACCACCCTGTTGCT |
| Convergent primers |  |  |
| OMA1 | Forward | AAGGCTGGCATGGTTCATTTG |
|  | Reverse | CTGTATCCACTGGCACAAAAGTG |
| RERE | Forward | AACTCCCAGGCCTGTTGC |
|  | Reverse | CCTCTCCCGGCTTCAGAAAG |
| GAPDH | Forward | GTCAGTGGTGGACCTGACCT |
|  | Reverse | TGACAAAGTGGTCGTTGAGG |
|  |  |  |
| siRNA | | |
| circ_14736 |  | 5'-3' |
| si-1 | sense | CCUCAGGGGCAUAAGGAAAUGTT |
|  | antisense | CAUUUCCUUAUGCCCCUGAGGTT |
| si-2 | sense | ACUUAUACCUCAGGGGCAUAATT |
|  | antisense | UUAUGCCCCUGAGGUAUAAGUTT |
| si-3 | sense | UACCUCAGGGGCAUAAGGAAATT |
|  | antisense | UUUCCUUAUGCCCCUGAGGUATT |
| circ_17720 |  |  |
| si-1 | sense | UGCACUGAACACAGUCCACAATT |
|  | antisense | UUGUGGACUGUGUUCAGUGCTT |
| si-2 | sense | GAAUGCACUGAACACAGUCCATT |
|  | antisense | UGGACUGUGUUCAGUGCAUUCTT |
| si-3 | sense | AAUGCACUGAACACAGUCCACTT |
|  | antisense | GUGGACUGUGUUCAGUGCAUUTT |

Table S1 Sequence of primers and siRNA used in this study
